# Peri-Procedural Fasting and Gastric Ultrasound Strategies in Glucagon-Like Peptide-1 (GLP-1) Receptor Agonist Users: A Systematic Review With Qualitative Synthesis

**DOI:** 10.7759/cureus.108216

**Published:** 2026-05-04

**Authors:** Osama Waleed Abdallah, Ahmad Albashtawi, Sara Aljabr, Nicole Issi, Abdelkader O Rababah, Nooran Alshyyab, Shams A Shadid, Aya N Alqurneh, Ahmad Difallah Al Darawsheh, Farah M Khazneh, Nuha Najib Almaghrebi, Abdelrahman K Alsaidi, Moneer Ibrahim I, Qais Shaban, Noor Rasheed, Mina Alhashemi, Majd Kharabsheh

**Affiliations:** 1 Internal Medicine, Prince Hamza Hospital, Amman, JOR; 2 Faculty of Medicine, University of Jordan, Amman, JOR; 3 Internal Medicine, King Hussein Medical Centre, Amman, JOR; 4 Faculty of Medicine, Jordan University Hospital, Amman, JOR; 5 Faculty of Medicine, Jordan University of Science and Technology, Irbid, JOR; 6 General Practice, Jameel Al Totangi Hospital, Amman, JOR; 7 Faculty of Medicine, Mutah University, Karak, JOR

**Keywords:** aspiration risk, fasting protocol, gastric ultrasound, glp-1 receptor agonist, peri-procedural management, point-of-care ultrasound, residual gastric content, semaglutide

## Abstract

Glucagon-like peptide-1 receptor agonists (GLP-1 RAs) delay gastric emptying, raising concerns about residual gastric content (RGC) and aspiration risk during elective procedures requiring anesthesia or sedation. RGC is a surrogate intermediate outcome that is distinct from clinical aspiration, which is a rarer event. This systematic review synthesizes available clinical evidence evaluating the association of peri-procedural mitigation strategies, including modified fasting protocols, medication withholding, and gastric point-of-care ultrasound (POCUS), with RGC reduction in GLP-1 RA users. A systematic search of PubMed, Embase, Cochrane CENTRAL, and Web of Science was conducted from inception to March 2026. Only original human studies were included; secondary research was excluded from the qualitative synthesis. Because study designs, populations, and outcome definitions differed substantially, findings were synthesized narratively at the study level; no meta-analysis was performed. Nineteen clinical studies, plus one administrative claims study, were analyzed separately and met the inclusion criteria. In one retrospective study, a 24-hour clear liquid diet was associated with lower RGC (approximately 1-2% versus 10%; odds ratio ≈ 0.13). In prospective ultrasound studies, withholding weekly GLP-1 RAs for 7-8 days was associated with lower odds of high RGC compared to shorter intervals. Multiple studies found that gastric POCUS using validated thresholds (>1.5 mL/kg or presence of solids) identified patients at increased risk. Aspiration events were rare and inconsistently reported across mitigation studies. The administrative claims study was analyzed separately as a distinct evidence tier and was not combined with clinical cohorts. Available observational evidence suggests that extended GLP-1 RA withholding, 24-hour clear liquid diets, and pre-procedural gastric POCUS may be associated with reduced RGC in selected patient populations. However, the evidence base remains limited, and a modest aspiration risk cannot be excluded. Large, prospective randomized trials are urgently needed.

## Introduction and background

Glucagon-like peptide-1 receptor agonists (GLP-1 RAs), including semaglutide, tirzepatide, liraglutide, and dulaglutide, have become widely prescribed for type 2 diabetes mellitus and obesity [1,2]. These agents slow gastric emptying as part of their therapeutic mechanism, which improves postprandial glycemic control and promotes satiety by prolonging nutrient delivery to the small intestine [3,4]. The delay in gastric emptying is mediated through vagal pathways and inhibition of antral motility, with GLP-1 RAs also suppressing glucagon secretion and enhancing glucose-dependent insulin release [3].

However, this pharmacologic effect raises significant safety concerns for patients undergoing elective procedures requiring anesthesia or sedation. Delayed gastric emptying increases the volume and alters the composition of residual gastric content (RGC), which in turn elevates the risk of pulmonary aspiration during induction of anesthesia, emergence, or intraprocedural periods [5]. Aspiration pneumonitis remains a leading cause of anesthesia-related morbidity, and any intervention that increases RGC volume above the traditional threshold of 0.4 mL/kg or introduces particulate matter is considered potentially hazardous.

Notably, RGC is a surrogate or intermediate outcome. A patient may have increased RGC on ultrasound or endoscopy without progressing to clinical aspiration, which depends on additional factors, including airway protection reflexes, anesthesia technique, and procedural positioning. This distinction is critical when interpreting the evidence base. This review focuses on evaluating peri-procedural mitigation strategies, including modified fasting protocols, medication withholding intervals, and gastric point-of-care ultrasound (POCUS) rather than re-establishing baseline risk. The objective is to qualitatively synthesize available clinical evidence from original human studies on which strategies are most effective at reducing RGC in GLP-1 RA users undergoing elective procedures requiring anesthesia or sedation.

## Review

Methods

Study Design

This study was designed as a systematic review and conducted in accordance with the Preferred Reporting Items for Systematic Reviews and Meta-Analyses (PRISMA) 2020 guidelines.

Search Strategy and Information Sources

A comprehensive and systematic literature search was conducted in PubMed, Embase, Cochrane CENTRAL, and Web of Science from database inception to March 2026. The search strategy was developed a priori and combined controlled vocabulary terms (Medical Subject Headings [MeSH], where applicable) with relevant free-text keywords related to GLP-1 RAs, gastric emptying, fasting protocols, gastric ultrasound, and aspiration to ensure comprehensive retrieval of eligible studies (Table 1).

**Table 1:** Search strategy by database

Database	Search Strategy	Filters	Results
PubMed	("GLP-1 receptor agonist"[MeSH] OR semaglutide[tiab] OR tirzepatide[tiab] OR liraglutide[tiab] OR dulaglutide[tiab]) AND ("gastric emptying"[MeSH] OR "residual gastric content"[tiab] OR "gastric ultrasound"[tiab] OR "POCUS"[tiab] OR aspiration[tiab] OR fasting[tiab])	Humans; Adults; English	312
Embase	('glucagon like peptide 1 receptor agonist'/exp OR semaglutide OR tirzepatide OR liraglutide OR dulaglutide) AND ('gastric emptying'/exp OR 'gastric content' OR 'gastric ultrasound' OR 'point of care ultrasound' OR aspiration OR fasting)	Humans; English	289
Cochrane CENTRAL	("GLP-1 receptor agonist" OR semaglutide OR tirzepatide OR liraglutide OR dulaglutide) AND ("gastric emptying" OR "residual gastric content" OR "gastric ultrasound" OR aspiration OR fasting)	Trials	98
Web of Science	TS=("GLP-1 receptor agonist" OR semaglutide OR tirzepatide OR liraglutide OR dulaglutide) AND TS=("gastric emptying" OR "gastric content" OR "gastric ultrasound" OR aspiration OR fasting)	English; Articles	148

Eligibility Criteria

Only original human studies in adults undergoing elective endoscopy, surgery, anesthesia, or sedation were included in the qualitative synthesis. Secondary literature, including systematic reviews, meta-analyses, scoping reviews, narrative reviews, guidelines, editorials, and consensus statements, was screened for reference mining only and excluded from synthesis. Eligible study designs included randomized controlled trials (RCTs), prospective cohort studies, retrospective observational studies, matched cohort studies, case-control studies, and case series with n ≥ 5. Case series with at least five patients were included as descriptive evidence only. Detailed inclusion and exclusion criteria are summarized in Table 2 according to the PICOS (Population, Intervention, Comparison, Outcome, Study Design) framework.

**Table 2:** Inclusion and exclusion criteria for study selection based on the PICOS framework PICOS: Population, Intervention, Comparison, Outcome, Study Design.

Category	Inclusion Criteria	Exclusion Criteria
Study Design	RCTs, prospective cohorts, retrospective cohorts, matched cohorts, case-control studies, case series (n ≥ 5)	Secondary research (systematic reviews, meta-analyses, scoping reviews, narrative reviews, guidelines), animal studies, in vitro research, case reports/series <5 patients, conference abstracts, editorials
Population	Adult patients (≥18 years) receiving GLP-1 RAs undergoing elective procedures (endoscopy or surgery) requiring anesthesia or sedation	Pediatric populations, healthy volunteers, non-procedural settings
Intervention	Peri-procedural mitigation strategies including modified fasting protocols, GLP-1 medication withholding, or gastric POCUS	Studies evaluating GLP-1 effects without a mitigation strategy
Outcomes	RGC (≥1.5 mL/kg or presence of solids by ultrasound or endoscopic visualization), gastric ultrasound findings, procedure cancellation, aspiration events	No relevant outcome data or absence of predefined outcomes
Other	English-language human studies with full-text availability	Abstracts-only, non-English, duplicates, insufficient methodological detail

Study Selection and Data Extraction

The initial database search identified 847 records. After removal of 134 duplicates, 713 studies underwent title and abstract screening, resulting in the exclusion of 651 records for irrelevance. Sixty-two full-text articles were sought for retrieval and assessed for eligibility. Of these, 43 reports were excluded based on the criteria above. A total of 19 clinical studies plus 1 administrative claims study (analyzed separately) met the inclusion criteria and were included in the qualitative synthesis.

**Figure 1:**
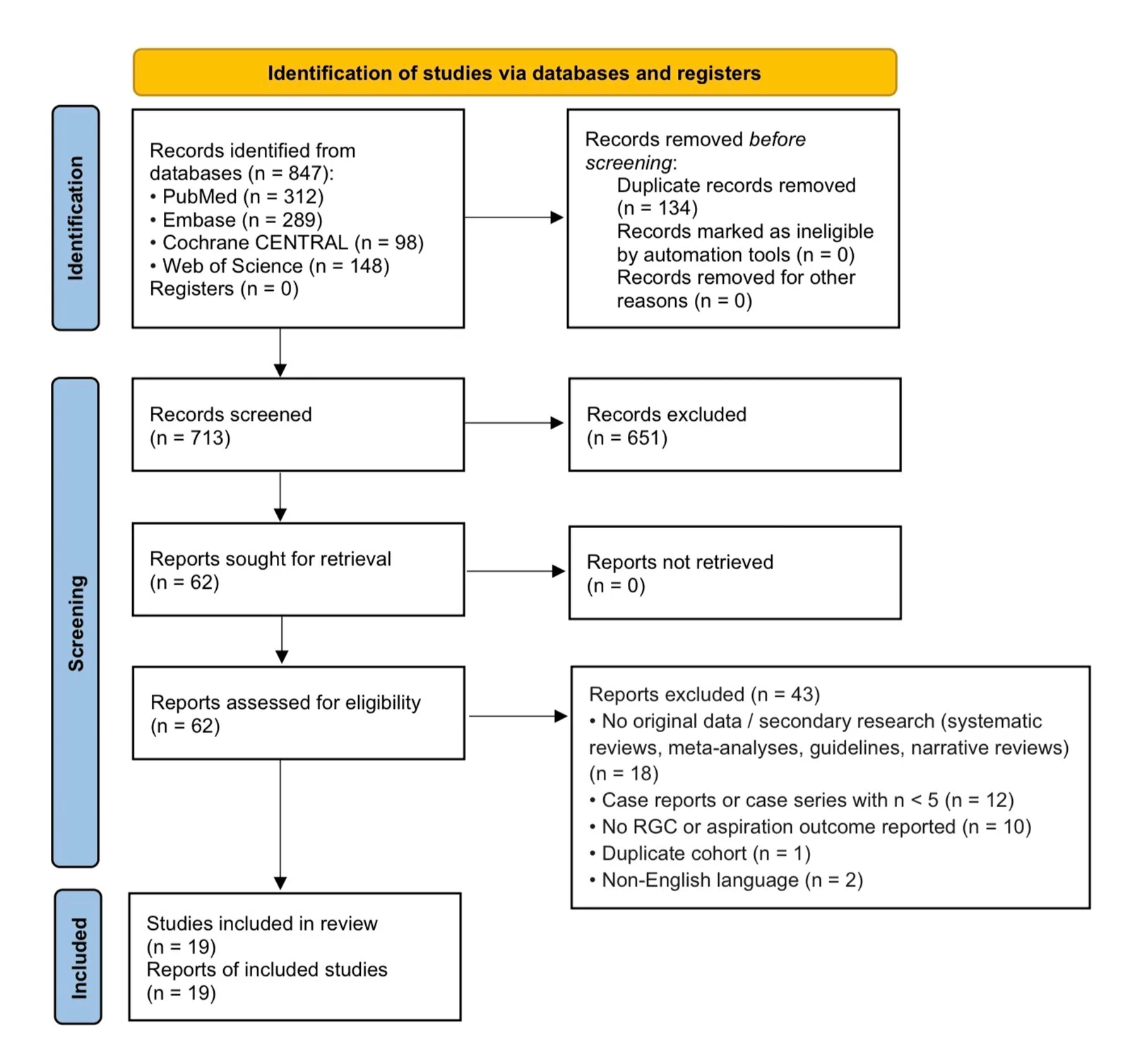
PRISMA flow diagram illustrating the study selection process for the systematic review PRISMA: Preferred Reporting Items for Systematic Reviews and Meta-Analyses.

Study screening (title/abstract and full-text) was performed independently by two reviewers (OWA and AA). Disagreements were resolved by consensus or consultation with a third reviewer (SA). Data extraction was performed independently by two reviewers (NI and AOR) using a standardized, piloted data extraction form. Inter-reviewer agreement for study inclusion was calculated using Cohen's kappa (κ = 0.89, 95% CI: 0.82-0.96), indicating near-perfect agreement. Discrepancies in data extraction were resolved through discussion. The selection process is illustrated in the PRISMA flow diagram (Figure 1).

Risk of Bias Assessment

Risk of bias was assessed independently by two reviewers using the Risk of Bias in Non-randomized Studies (ROBINS-I) tool for non-randomized studies and the NIH Quality Assessment Tool for observational cohort and cross-sectional studies. Disagreements were resolved by consensus. Studies were rated as low, moderate, or serious risk of bias based on confounding, selection bias, measurement of outcomes, and completeness of follow-up. Prospective and adjusted studies were weighted more heavily in interpretation than case series or single-center retrospective studies. Results of the risk of bias assessment are summarized in Table 3.

**Table 3:** Risk of bias assessment of included studies ROBINS-I = Risk of Bias in Non-randomized Studies, NIH = National Institute of Health.

Study	Study Design	ROBINS-I / NIH Tool	Overall
Wu et al., 2024 [6]	Retrospective cohort	Serious	Serious
Silveira et al., 2023 [7]	Retrospective cohort	Moderate	Moderate
Abu-Freha et al., 2024 [8]	Retrospective cohort	Moderate	Moderate
Hisada et al., 2025 [9]	Matched cohort	Moderate	Moderate
Ghazanfar et al., 2024 [10]	Retrospective cohort	Serious	Serious
Maselli et al., 2024 [11]	Case series	Moderate	Moderate
Nersessian et al., 2024 [12]	Prospective cohort	Low	Low
Wolla et al., 2025 [13]	Prospective cohort	Low	Low
Vlaeminck et al., 2026 [14]	Prospective cohort	Low	Low
Pai et al., 2026 [15]	Prospective cohort	Low	Low
Sen et al., 2024 [16]	Prospective cohort	Low	Low
Alkabbani et al., 2024 [17]	Retrospective cohort	Moderate	Moderate
Mahmoud et al., 2025 [18]	Prospective cohort	Low	Low
Kobori et al., 2023 [19]	Retrospective cohort	Moderate	Moderate
Stark et al., 2022 [20]	Retrospective cohort	Moderate	Moderate
Santos et al., 2024 [21]	Retrospective cohort	Moderate	Moderate
Panchal et al., 2025 [22]	Retrospective cohort	Moderate	Moderate
Queiroz et al., 2024 [23]	Cross-sectional	Low	Low
Shahidifar et al., 2025 [24]	Prospective cohort	Low	Low
Wright et al., 2025 [25]	Retrospective claims	Moderate	Moderate

Data Synthesis

Because the included studies differed substantially in design, procedure type, GLP-1 agent, outcome definition, and withholding interval, findings were synthesized narratively at the study level. No meta-analysis was performed, and no estimates were pooled. Studies were grouped by mitigation strategy (modified fasting protocols, medication withholding, gastric POCUS) and then by outcome type (RGC, full stomach by ultrasound, aspiration events). Effect size estimates (e.g., ORs, percentage reductions) were extracted where available and are reported as presented in each original study. Results are reported at the individual study level only. The administrative claims database study [25] was retained as a separate evidence tier and was not combined with clinical studies when summarizing findings.

Ethical Considerations

Ethical approval and informed consent were not required, as this review analyzed data exclusively from previously published studies.

Results

Study Characteristics

Nineteen clinical studies examining GLP-1 RA mitigation strategies met inclusion criteria, comprising prospective ultrasound cohorts, retrospective endoscopic cohorts, matched cohort studies, and case series. One additional administrative claims database study is presented separately as a distinct evidence tier. Tables 4, 5 present study characteristics separated by evidence tier [25].

**Table 4:** Clinical studies (prospective and retrospective cohorts, case series) EGD = esophagogastroduodenoscopy; RGC = residual gastric content; POCUS = point-of-care ultrasound; OR = odds ratio; GI = gastrointestinal; ESG = endoscopic sleeve gastroplasty; LOS = length of stay; NS = not significant.

Study (year)	Study design	Sample size (GLP-1 / total)	Population / setting	GLP-1 agent	Procedure / mitigation strategy	Key finding
Stark et al., 2022 [20]	Retrospective cohort	Not fully reported	EGD	Not specified	Standard fasting	Increased food retention
Silveira et al., 2023 [7]	Retrospective cohort	33/404	Elective EGD	Semaglutide	Standard fasting vs withholding	RGC: 24.2% vs 5.1%; OR 5.15
Kobori et al., 2023 [19]	Retrospective cohort	Not fully reported	EGD	Not specified	Standard fasting	Increased gastric residue
Maselli et al., 2024 [11]	Case series	57/57	Endoscopic sleeve gastroplasty	Not specified	24h solids / 12h liquids	No retained solids; 0% aspiration
Wu et al., 2024 [6]	Retrospective cohort	90/192	Elective EGD under anesthesia	Not specified	Standard fasting	RGC: 19% vs 5%; OR 5.8
Abu-Freha et al., 2024 [8]	Retrospective cohort	1,671/120,879	Upper endoscopy	Not specified	Standard fasting	RGC: 5.6% vs 2.0%
Ghazanfar et al., 2024 [10]	Retrospective cohort	156/312	EGD	Not specified	24h clear liquid diet	RGC: ~1–2% vs 10%
Nersessian et al., 2024 [12]	Prospective cohort	107/220	Elective surgery	Semaglutide	POCUS; interruption	RGC: 40% vs 3%
Sen et al., 2024 [16]	Prospective cohort	Not fully reported	Pre-anesthesia	Not specified	POCUS	Increased RGC
Alkabbani et al., 2024 [17]	Retrospective cohort	Not fully reported	Upper GI endoscopy	Not specified	None	No increase in aspiration
Santos et al., 2024 [21]	Retrospective cohort	Not fully reported	EGD	Not specified	Withholding	Protective
Queiroz et al., 2024 [23]	Cross-sectional	30 total	Bariatric surgery candidates	Semaglutide	Ultrasound	73% vs 7%
Wolla et al., 2025 [13]	Prospective cohort	106/206	Elective surgery	Not specified	POCUS; timing	OR 11.3
Hisada et al., 2025 [9]	Matched cohort	148/296	EGD	Not specified	Standard fasting	12.2% vs 3.4%
Mahmoud et al., 2025 [18]	Prospective cohort	426 total	Orthopedic surgery	Not specified	None	0% aspiration
Panchal et al., 2025 [22]	Retrospective cohort	Not fully reported	Outpatient endoscopy	Not specified	POCUS	OR ≈ 3.8
Vlaeminck et al., 2026 [14]	Prospective cohort	44/88	Elective surgery	Semaglutide	POCUS	OR 9.85
Pai et al., 2026 [15]	Prospective cohort	316/316	Pre-anesthesia	Not specified	POCUS; withholding	35.8% high RGC
Shahidifar et al., 2025 [24]	Prospective cohort	Not fully reported	Pre-anesthesia	Not specified	Ultrasound; fasting duration	Fasting duration more protective than GLP-1 timing alone

**Table 5:** Administrative database study (separate evidence tier) Administrative database studies are presented separately from clinical cohorts because outcome ascertainment (diagnosis codes versus direct visualization), confounding control, and generalizability differ substantially. These estimates should not be pooled or directly compared with clinical study findings.

Study (year)	Study Design	Population	Sample Size	Key Outcome	Effect Estimate
Wright et al., 2025 [25]	Retrospective claims database	Elective surgery	15,745/392,065	Aspiration	OR 1.07 (95% CI: 0.85-1.34, NS)

Effects of mitigation strategies

Modified Fasting Protocols

In a retrospective study, a 24-hour clear-liquid diet was associated with residual food in approximately 1-2% of the diet group compared to 10% of the standard fasting group (OR ≈ 0.13) [10]. No aspiration events were reported. Another study evaluated a protocol of 24-hour solid fasting and 12-hour liquid fasting in 57 patients undergoing endoscopic sleeve gastroplasty, observing no retained solids or aspiration [11]. In a prospective ultrasound study, prolonged fasting duration was reported to be more protective against high gastric volume than the timing of the last GLP-1 dose alone [24].

Medication Withholding

Multiple studies have examined optimal GLP-1 RA withholding intervals. In a prospective ultrasound study, GLP-1 RA use within seven days of surgery was associated with significantly higher gastric volumes (OR 11.3, 95% CI: 5.2-24.7, p < 0.001) compared with holding therapy for over seven days [13]. Another study [15] identified a receiver operating characteristic (ROC)-derived cut-off of ≥7.5 days for medication withholding as protective against high RGC. A prospective study reported that semaglutide use within 10 days of surgery was associated with 40% RGC compared to 3% in controls (OR 36.97, p < 0.001) [12]. In a retrospective study, semaglutide interruption intervals were associated with RGC (OR 5.15, 95% CI: 1.92-12.92, p < 0.01) [7]. Another retrospective study similarly reported that GLP-1 withholding was protective against RGC [21]. Evidence for additional benefit beyond 14 days remains limited.

Gastric Point-of-Care Ultrasound (POCUS)

Gastric POCUS allows real-time risk stratification. In GLP-1 RA users specifically, multiple prospective studies have demonstrated utility. One study reported an odds ratio of 9.85 (95% CI: 2.57-37.76) for the presence of solids in semaglutide users compared to matched controls [14]. Another prospective study evaluated pre-anesthesia patients and found significantly higher RGC volumes in GLP-1 users [16]. A cross-sectional study demonstrated that 73% of semaglutide users had a "full stomach" by ultrasound criteria compared to 7% of controls (p < 0.001) [23]. Another retrospective study reported an odds ratio of approximately 3.8 for increased RGC in GLP-1 users undergoing outpatient endoscopy [22]. The prospective study by Shahidifar et al. further highlighted that longer fasting duration, rather than just GLP-1 timing, was associated with lower gastric volumes [24].

Aspiration Outcomes

Aspiration events were rare across all included studies. In a prospective study of 426 GLP-1 users undergoing orthopedic surgery, zero aspiration events were reported, though the length of stay was increased [18]. Another study found no increase in aspiration in GLP-1 users undergoing upper gastrointestinal (GI) endoscopy [17]. In the administrative claims database study (presented as a separate evidence tier), aspiration occurred in 0.8% of GLP-1 RA users versus 0.7% of controls (adjusted OR = 1.07, 95% CI: 0.85-1.34, not significant) [25].

Discussion

This systematic review provides a qualitative synthesis of clinical evidence from 19 clinical studies (plus one administrative claims study analyzed separately) evaluating peri-procedural mitigation strategies in GLP-1 RA users. Across individual studies, GLP-1 RAs were consistently associated with increased residual gastric content (RGC) compared to non-users, although this risk appears modifiable with targeted interventions. RGC is a surrogate outcome distinct from clinical aspiration, and these outcomes were interpreted separately. Increased RGC does not necessarily translate to aspiration, which depends on additional factors, including airway reflexes, anesthesia technique, and procedural context.

Modified fasting protocols showed potential benefit. One retrospective study demonstrated that a 24-hour clear-liquid diet reduced RGC to approximately 1-2% compared to 10% with standard fasting [10]. A case series reported no retained solids or aspiration with extended fasting [11]. A prospective ultrasound study further suggested that fasting duration may be more protective than GLP-1 timing alone [24].

Medication withholding was consistently associated with lower RGC in multiple studies. Prospective ultrasound data [12,13,15] indicate that withholding GLP-1 RAs for approximately seven to eight days reduces the likelihood of high RGC compared to shorter intervals. These findings support partial reversibility of drug-induced gastric stasis, although optimal intervals may vary by formulation and patient characteristics. Gastric POCUS provides real-time risk stratification and demonstrated consistent utility across studies [12-16,22-24]. Validated thresholds (>1.5 mL/kg or presence of solids) allow identification of patients at higher risk and may inform procedural decision-making, including delay or modification of anesthesia approach.

Aspiration events were rare across all studies. Cohort data did not demonstrate a statistically significant increase in aspiration risk, although studies were likely underpowered for this outcome. In the administrative claims study [25], aspiration occurred in 0.8% of GLP-1 users versus 0.7% of controls (adjusted OR 1.07, 95% CI: 0.85-1.34). The confidence interval includes a possible modest increase in risk. Prospective studies reported zero aspiration events, but sample sizes (e.g., n=426 [18]) are insufficient to exclude clinically meaningful differences given the low baseline incidence (~0.1-0.4%). The observed dissociation between RGC and aspiration likely reflects low baseline risk, cautious perioperative management, selective case postponement, and possible underdetection of mild events. This review excluded secondary research from the primary synthesis; therefore, findings reflect only individual original studies and are not directly comparable to pooled analyses.

Limitations and future directions

Several limitations should be considered. Substantial heterogeneity existed across study design, population, GLP-1 agent, fasting protocols, withholding intervals, and outcome definitions, precluding meta-analysis and limiting generalizability. The number of prospective studies was small, and several retrospective studies carried a moderate-to-serious risk of bias with incomplete reporting. Case series were included as descriptive evidence but lack comparators. Publication bias cannot be excluded. No sensitivity analyses were performed due to limited study numbers. Administrative database studies differ fundamentally from clinical cohorts in outcome ascertainment and were analyzed separately. Finally, all included studies were observational, and residual confounding remains likely. Large, well-designed prospective randomized trials are needed to determine optimal withholding intervals, evaluate the incremental benefit of modified fasting, assess POCUS-guided strategies, and define the true absolute risk of aspiration associated with GLP-1 RA use.

## Conclusions

Available observational evidence suggests that 24-hour clear liquid diets, longer GLP-1 RA withholding intervals (approximately 7-8 days), and pre-procedural gastric POCUS may be associated with reduced RGC in selected patient populations undergoing elective procedures. Large cohort studies have not shown a statistically significant increase in aspiration, but rare-event limitations prevent firm conclusions, and a modest aspiration risk cannot be excluded. Heterogeneity and methodological limitations of existing studies prevent definitive clinical recommendations. Future large-scale, well-designed prospective trials focusing on standardized mitigation protocols are warranted.
